# Highlights from Heart Rhythm Society 2017: Innovations in Electrophysiology Patient Management, and Beyond

**DOI:** 10.19102/icrm.2017.080804

**Published:** 2017-08-15

**Authors:** Sugrue Alan, Vaibhav Vaidya, Samuel Asirvatham


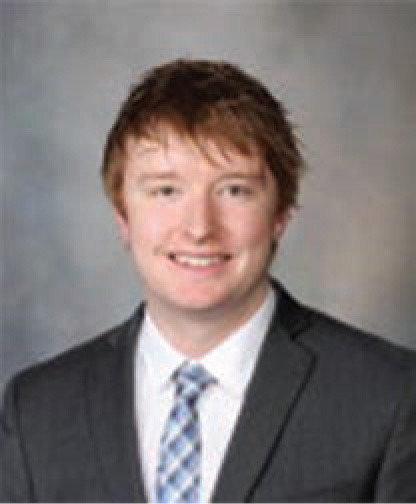



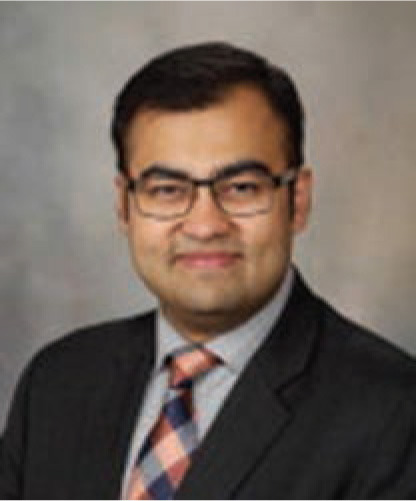



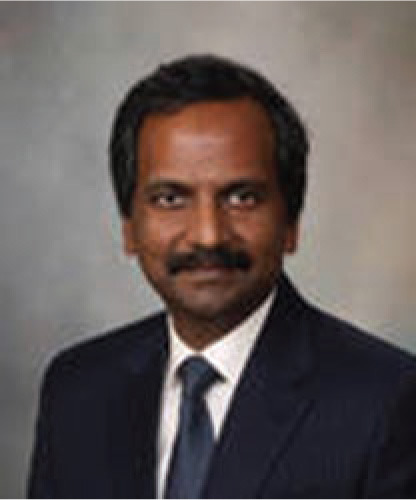


## Introduction

In the following article, we share our insights from the exceedingly successful 2017 Heart Rhythm Society’s (HRS) 38th Annual Scientific Sessions meeting, held in May 2017 in Chicago, IL. Below, we have outlined research abstracts from a range of different presentation formats that we feel are noteworthy and that represent some of the biggest takeaways from HRS 2017.

### Late-breaking clinical trials

***Frequency of atrial fibrillation (AF) in at-risk patients revealed.*** AF is the most common arrhythmia worldwide, and is known to be associated with stroke. Anticoagulation can decrease the risk of stroke in patients with AF. The increasing use of cardiovascular implantable electronic devices (CIEDs) and long-term rhythm monitoring technologies has enabled the detection of brief episodes of AF. Studies in patients without a prior history of AF and CIEDs showed that greater than six minutes of AF is associated with an elevated risk of stroke.^1^ Long-term monitoring performed in patients with cryptogenic stroke and no prior history of AF showed a high incidence of previously unrecognized or silent AF.^2,3^

The REVEAL AF study was a prospective multicenter study designed to determine the incidence of AF ≥ six minutes in length in patients at high risk (CHADS2 score ≥ 3, or = 2 with an additional risk factor); but without a known history of AF.^10^ In this study, an implanted cardiac monitor (ICM) was used to detect AF. At 18 and 30 months, 29.3% and 40% of patients had AF. Median time-to-detection of first AF episode was 123 days. This study confirms a similarly high incidence of background AF in at-risk patients as has been reported in other studies examining the use of an ICM in at-risk patients (such as the PREDATE AF^4^ and ASSERT II^5^ studies).

These studies raise important questions about our current understanding of stroke and stroke prevention in AF. In the CRYSTAL AF and EMBRACE studies, patients with cryptogenic stroke underwent long-term rhythm monitoring with ICMs versus usual care. AF was detected in 12.4% and 16.1% of patients with ICMs, compared with 2% and 3.2% of usual care patients in these studies, respectively. There appeared to be a significant incidence of previously unrecognized AF, which could be the etiology of cryptogenic stroke in these patients. However, PREDATE AF, ASSERT II, and, now, REVEAL AF, demonstrate that the background rate of AF is high even in patients without an obligatory history of stroke. Could this mean that AF is an epiphenomenon and not the cause of stroke in cryptogenic stroke patients?

On the other hand, it is possible that AF detected by ICM does increase stroke in the long term. The risk might become apparent as the PREDATE AF, ASSERT II, and REVEAL AF cohorts are followed long term. Can anticoagulation for ICM-detected silent AF reduce stroke risk significantly? The ongoing NOAH (https:// clinicaltrials.gov/ct2/show/NCT02618577) and ARTESiA (https://clinicaltrials.gov/ct2/show/NCT01938248) trials are expected to provide much-needed information regarding the modification of stroke risk in patients with incidentally detected AF.

***What is the optimal antiarrhythmic drug strategy after AF ablation?*** Catheter ablation for AF is increasingly used following antiarrhythmic drug failure, or as first-line therapy in patients with paroxysmal AF. The duration of antiarrhythmic drug therapy following catheter ablation is inconsistent between patients, with some operators and centers continuing antiarrhythmic drugs for varying lengths of time, and others discontinuing the drugs in patients in sinus rhythm.

In a multicenter randomized trial (POWDER AF), the authors compared two antiarrhythmic drug strategies applied following ablation for paroxysmal AF.^11^ Previously ineffective antiarrhythmic drug therapy was continued for three months post ablation, after which patients in sinus rhythm were randomized to drug continuation versus discontinuation. The primary outcome of any documented tachyarrhythmia > 30 s occurred in two of 74 patients in the drug continuation arm, and in 16 of 73 patients in the drug discontinuation arm. The drug continuation arm had lower rates of repeat ablation (1.3% versus 17.1%) and unscheduled visits (2.6% versus 19.7%) than the drug discontinuation arm did, with similar quality of life (QOL) scores noted between the two groups.

This is a valuable study to facilitate shared decision-making between physicians and patients with paroxysmal AF undergoing ablation. For patients who tolerate antiarrhythmic therapy and who aim to be free of AF, antiarrhythmic drug therapy greatly improves the likelihood of achieving this goal. Patients who do not tolerate antiarrhythmic drugs can be counseled regarding the magnitude of increased arrhythmia recurrence risk present while off drug therapy.

### Featured abstracts

***Does a delay in anticoagulation initiation increase the risk of dementia among AF patients?*** Bunch et al. presented an intriguing observational study that involved a large cohort of 26,189 AF patients without a history of dementia.^17^ The study participants were stratified into immediate (< 30 days) and delayed (> 30 days) initiation of anticoagulant or antiplatelet therapy from their dates of AF diagnosis. Delayed initiation of anticoagulation was associated with an increased risk of incident dementia on multivariate analysis (hazard ratio (HR): 1.63, p = 0.05). The relative risk increased with increasing CHADS2-VASc scores (0–1 HR: 1.3, p = 0.75; 2–4 HR: 1.5, p = 0.19; > 5 HR: 2.36, p = 0.07).

This study reports on the association of dementia with delayed initiation of therapy for AF, but causation is uncertain due to the observational nature of the study. There might be unmeasured or unidentified confounding factors that could lead to this association. The authors have recently initiated a study comparing dabigatran to warfarin for anticoagulation in AF, evaluating the primary outcome of incident dementia (https://clinicaltrials.gov/ct2/show/NCT03061006). The results of this study are expected to be important in guiding the selection of anticoagulant choice in AF patients.

### Poster presentations

***Inappropriate sinus tachycardia—is exercise a way forward?.*** Inappropriatesinus tachycardia (IST) is a debilitating disease with a spectrum of symptoms that can have devastating effects on an individual’s way of life The pathophysiology of IST remains poorly understood, but is believed to be multifactorial. At present, there are limited options for treatment, and the current options that are available, such as ablation, are fraught with potentially disastrous complications (including superior vena cava syndrome and right diaphragmatic paralysis), and have only shown modest long-term outcomes.^6^

In this noteworthy abstract, a small randomized trial that examined the role of exercise training in addition to pharmacology therapy (metoprolol) presented intriguing data which seems to hold some promise for the treatment of this debilitating condition.^12^ In the study, among those patients enrolled in the exercise training group, 80% reported significant and stable improvements in their perception of their exercise tolerance, as well as in total QOL, which was associated with a decrease in IST-related symptoms.

Further studies are required, but this seems to be a promising treatment option to look into further, and is one that would be easy to counsel patients about.

***Abandoned leads and magnetic resonance imaging—safe to use?.*** Historically,magnetic resonance imaging (MRI)-based examinations of patients with CIEDs have been considered hazardous to perform However, a number of observational studies have reported that MRI scanning can be performed safely in carefully chosen patients who have implanted devices. More recently, the MagnaSafe study^7^ showed that device or lead failure did not occur in any patients with non-MRI-conditional pacemakers or implanted cardioverter-defibrillators who underwent clinically indicated non-thoracic MRI scanning at 1.5 tesla. However, there was an exclusion of those patients with abandoned leads and those who were pacing dependent from this study.

Three posters at HRS 2017 addressed the use of MRI in patients with devices and abandoned leads.^13–15^ Across all three studies, there were 92 patients with 117 MRI scans included, with all showing that there was no clinical or electrical evidence of any CIED dysfunction, and no occurrence of arrhythmia or pain during the scan. These data show significant promise, and we look forward to reading these completed manuscripts to see details on the inclusion and exclusion criteria.

For the time being, however, it is important to note that the 2017 HRS Expert Consensus Statement Devices (published at the time of HRS2017) advises against MRI scans being done in patients with abandoned leads.^8^

### Device technology

***Painless cardioversion—yes please!.*** AFis a growing epidemic and, as there are increasing indications for device implantation, clinicians are bound to encounter patients with both AF and a device With the device in place, it would seem logical that it could be used to facilitate cardioversion. However, at present, this often requires a painful shock (as the AF threshold remains above the pain threshold). There have been previous publications involving animal models with the use of low-energy multistage electrotherapy, which all have shown significant promise. Now, we have the first reported results involving human subjects.

Sixteen patients were recruited for participation in this study, with 13 patients ultimately undergoing electrically induced AF.^16^ The authors showed multistage electrotherapy (MSE) converted AF in nine of the 13 patients (70%), with a lowest energy mean value of 0.57 ± 0.56 J. The authors do comment on factors observed that limited MSE, including the dislodgement of leads and coronary sinus anatomy. With further work and refinement of this technique, it could offer a significant option for device-based cardioversion in the future.

### Consensus statements

***New expert consensus statement on catheter and surgical ablation of AF—keeping up with rapid evolution.*** Ina joint partnership with the European Heart Rhythm Association (EHRA), the European Cardiac Arrhythmias Society (ECAS), the Asia Pacific Heart Rhythm Society (APHRS), and the Sociedad Latinoamericana de Estimulación Cardíaca y Electrofisiología (SOLAECE), HRS presented and published an updated consensus statement which presented a state-of-the-art review on the field of catheter and surgical ablation of AF.^9^ This consensus document is the third iteration on this subject, with the first and second incarnations issued in 2007 and 2012, respectively. The 2017 version summarizes the opinion of the writing group members (which included 60 individuals from different geographic locations) based on the results of an extensive literature review, as well as their own experience, and clearly states that it is not meant to represent a formal guideline.

That being said, however, given how fast the field of ablation is evolving, this document still represents an important body of work and is extensive in its information (270 pages). While we encourage readers to review it closely, there are a few recommendations in particular that we would like to point out.

*Ablation of asymptomatic AF.* While catheter ablation is an established treatment for patients who are symptomatic and either refractory to anti-arrhythmia therapy (Class I recommendation), or as first-line upfront therapy (Class IIa), this consensus statement specifically tried to address the issue of catheter ablation in asymptomatic patients. This document states that after careful discussion of the risks, benefits and alternatives, AF ablation may be considered in select asymptomatic patients with paroxysmal or persistent AF when performed by an experienced operator (Class IIb, LOE C-EO). Although evidence is relatively sparse, the rationale for this recommendation is that the amount of time a patient is in continuous AF directly impacts the outcomes of AF ablation or other rhythm control strategies, and it is plausible that achieving sinus rhythm may reduce the risk of stroke, heart failure and/or dementia. Notably, further data are still needed on these patients, but it is an area to watch and observe what data are presented on this subject in the future.

*Anticoagulation.* Periprocedural anticoagulation is of major benefit in reducing thromboembolism (TE) events. While historically, warfarin was held and the patient was “bridged” (ie, warfarin therapy was interrupted for a period of time), this was shown to elicit significantly more bleeding and TE events than in those who were continued on warfarin uninterrupted. Newer evidence regarding the use of novel anticoagulants (eg, dabigatran, rivaroxaban, apixaban, and edoxaban) in this situation has been published. The writing committee for the current consensus statement believes that the data and worldwide experience to date with novel oral anticoagulants are now sufficient to provide a Class I recommendation for performing AF ablation with uninterrupted dabigatran (Class I, LOE-A) or rivaroxaban (Class I, LOEB-R), and a Class IIa recommendation for the other Xa inhibitors.

***More on MRI.*** Asmentioned before, a second notable document, the “2017 HRS Expert Consensus Statement on Magnetic Resonance Imaging and Radiation Exposure in Patients with Cardiovascular Implantable Devices,” was also announced during the meeting.^8^ Eleven societies (the American College of Cardiology, the American College of Radiology, the American Heart Association, the Asia Pacific Heart Rhythm Society, the American Society for Radiation Oncology, the Council of Affiliated Regional Radiation Oncology Societies, the European Heart Rhythm Association, the Japanese Heart Rhythm Society, the Pediatric and Congenital Electrophysiology Society, Sociedade Brasileira de Arritmias Cardíacas, and Sociedad Latinoamericana de Estimulación Cardíaca y Electrofisiología) contributed to the development of this guideline document, which seeks to describe detailed practical recommendations for health-care providers to follow when working with patients with CIEDs who require cardiac mapping or treatments that may necessitate the use of an MRI environment. Included in the document is information on the risk relationship between

MRI technology and CIED systems; the definition and use of MRI-conditional CIED technology; the management of patients with CIEDs referred for an MRI or computed tomography scan, or radiation therapy; and suggested provisions for institutional protocols for the magnetic resonance scanning of patients with CIEDs.

## Conclusion

Though listed here are some of the most interesting items to come out of HRS 2017, this list is by no means exhaustive. We look forward to the continued release of research results and data on these topics and others, and to next year’s meeting.

Alan Sugrue, MD

Sugrue.Alan@mayo.edu

Department of Cardiovascular

Diseases

Division of Heart Rhythm Services

Mayo Clinic

Rochester, MN 55905

Vaibhav Vaidya, MD

Vaidya.Vaibhav@mayo.edu

Department of Cardiovascular

Diseases

Division of Heart Rhythm Services

Mayo Clinic

Rochester, MN 55905

Samuel Asirvatham, MD

Asirvatham.Samuel@mayo.edu

Department of Cardiovascular

Diseases

Division of Heart Rhythm Services

Department of Pediatric and

Adolescent Medicine

Division of Pediatric Cardiology

Mayo Clinic

Rochester, MN 55905
